# Pain prevalence and intensity in advanced pancreatic cancer: a nationwide cohort study

**DOI:** 10.1097/j.pain.0000000000004059

**Published:** 2026-07-06

**Authors:** Aniek E. Van Diepen, Merlijn U.J.E. Graus, Simone Augustinus, Anne M. Gehrels, Pauline A.J. Vissers, Eline H.E. van Gansewinkel, Marc G. Besselink, Hanneke W.M. van Laarhoven, Stefan A.W. Bouwense, Johanna W. Wilmink, Geert A. Cirkel, Nigel G. Kooreman, Ricardo Alvarez Jimenez, Jan van Zundert, Marjolein Y.V. Homs, Sander M.J. van Kuijk, Judith de Vos-Geelen

**Affiliations:** a Division Medical Oncology, Department Internal Medicine, GROW—Research Institute for Oncology and Reproduction, Maastricht University Medical Centre, Maastricht, the Netherlands; b Department of Medical Oncology, Erasmus MC Cancer Institute, Rotterdam, the Netherlands; c Solid Tumour Immunology Research Rotterdam (STIRR) Group, Department of Pulmonary Medicine, Erasmus University Medical Centre, Rotterdam, The Netherlands; d Department of Surgery, Amsterdam UMC, Location University of Amsterdam, Amsterdam, the Netherlands; e Department of Medical Oncology, Amsterdam UMC, Location University of Amsterdam, Amsterdam, the Netherlands; f Cancer Center Amsterdam, Cancer Treatment and Quality of Life, Amsterdam, the Netherlands; g Department of Research and Development, Netherlands Comprehensive Cancer Organization (IKNL), Utrecht, the Netherlands; h Department of Surgery, Radboud University Medical Center, Nijmegen, the Netherlands; i Cancer Center Amsterdam, Cancer Treatment and Quality of Life, the Netherlands; j Department of Surgery, Maastricht University Medical Centre, Maastricht, the Netherlands; k NUTRIM School of Nutrition and Translational Research in Metabolism, Maastricht University, Maastricht, the Netherlands; l Department of Medical Oncology, Regional Academic Cancer Centre Utrecht, St. Antonius Hospital, University Medical Centre Utrecht, Utrecht, the Netherlands; m Department of Surgery, Erasmus MC, University Medical Centre Rotterdam, Rotterdam, the Netherlands; n Department of Anesthesiology and Pain Management, Amsterdam UMC, Location University of Amsterdam, Amsterdam, the Netherlands; o Department of Anesthesiology and Pain Management, Maastricht University Medical Centre, Maastricht, the Netherlands; p Division Translational Neuroscience, Mental Health and Neuroscience Research Institute, Maastricht University, the Netherlands; q Department of Anesthesiology, Intensive Care, Emergency Medicine and Multidisciplinary Pain Center, Ziekenhuis Oost-Limburg, Belgium; r Department of Clinical Epidemiology and Medical Technology Assessment, Maastricht University Medical Centre, Maastricht, the Netherlands

**Keywords:** Supportive care, Symptom, Pain, Discomfort, Pancreas, Carcinoma, Metastatic, Locally advanced pancreatic cancer

## Abstract

Pain is a major concern in patients with advanced pancreatic cancer, significantly affecting quality of life, treatment tolerance, and end-of-life experience. However, data on its prevalence, course, and determinants remain limited, particularly regarding the dynamics of pain as a primary outcome in real-world patient populations. This nationwide cohort study, conducted within the PACAP-PROM registry (2016-2023), included patients with locally advanced (LAPC) or metastatic pancreatic cancer (mPC). Pain was assessed using 5 items from the EORTC QLQ-C30, PAN26, and EQ-5D-5L questionnaires, with the primary outcome being pain prevalence at baseline and comparisons between LAPC and mPC. Secondary outcomes included pain trajectories over 3 to 18 months and the association between chemotherapy and pain, evaluated using mixed-effects logistic regression. Overall, 794 patients were included, of whom 648 (82%) received cancer therapy, and 146 (18%) received best supportive care (BSC). At baseline, 54% to 78% of patients reported pain, with no differences between LAPC and mPC. Although pain prevalence decreased at 3 months, variable patterns developed thereafter. Patients receiving BSC reported more pain (57% vs 38%, *P* = 0.029) and greater pain-related interference with daily activities (53% vs 38%, *P* = 0.024). Chemotherapy was independently associated with lower odds of higher pain severity categories for current pain (OR 0.51; 95% CI 0.37-0.70) and pain interference (OR 0.44; 95% CI 0.31-0.63). In conclusion, pain prevalence exceeds 50% in patients with advanced pancreatic cancer and remains a key concern that can be positively influenced by chemotherapy. These findings emphasize the need for routine pain assessment and individualized symptom management from diagnosis onward.

## Abstract 2

Supplemental Digital Content is Available in the Text.

Pain affects over 50% of advanced pancreatic cancer patients and can be reduced by chemotherapy, highlighting the need for regular pain assessments and personalized management.

## 1. Introduction

Pain is one of the most common and debilitating symptoms of pancreatic cancer, affecting up to 80% of patients overall and 90% in advanced stages.^6,9,13,38,40,42^ The underlying mechanisms are multifactorial and include pancreatic neuropathy, autonomic plexus invasion, perineural spread, and metastatic involvement of surrounding tissues.^4,6,9,25,42^ If inadequately managed in this setting, pain can severely compromise quality of life (QoL), interfere with patients' tolerance to treatment, and limit opportunities for shared decision-making and advance care planning.^7,44,54^

Current national guidelines recommend opioids as first-line treatment for pancreatic cancer pain.^20,49^ However, when pain becomes refractory to pharmacological management, more invasive interventions, such as celiac plexus or splanchnic nerve blocks, radiotherapy, or pulsed radiofrequency lesioning, may be required.^19,39,40,48,52,60^ These interventions are typically reserved for later stages of disease, when patients already face a high symptom burden and limited physiological reserve.^17^ Given the potential side effects of opioids and the burden of interventional procedures, proactive and well-timed pain management is essential.^8,20^

In patients with locally advanced or metastatic pancreatic cancer, chemotherapy is a cornerstone of treatment and is primarily used to prolong survival. However, emerging evidence suggests that chemotherapy may also alleviate cancer-related pain, a symptom with substantial impact on quality of life. Pain relief with chemotherapy may result from tumor shrinkage, reduced neural invasion, or decreased peritumoral inflammation.^46^ This potential benefit is clinically relevant because the survival improvement is modest and symptom control often becomes the primary therapeutic goal.^33,44,50^ However, the progression of pain and its development during systemic treatment remain poorly researched. In addition, specific pain phenotypes such as breakthrough cancer pain (BTcP) have been described in cancer populations, although pancreatic cancer–specific data remain limited.^13,55^ Pain is typically evaluated as a secondary endpoint in larger QoL studies,^22,33,46,50,53,57,58^ leaving clinicians without clear guidance on its long-term course or how systemic therapy might affect it. There is a shortage of high-quality, prospective data that focuses explicitly on pain as the primary outcome.

Therefore, this study aimed to assess the prevalence and evolution of patient-reported pain over time and to explore the potential analgesic effects of chemotherapy in both locally advanced (LAPC) and metastatic pancreatic cancer (mPC).

## 2. Methods

### 2.1. Study design and data collection

This was a retrospective analysis of prospectively collected multicenter cohort data from the Dutch Pancreatic *Cancer* Project (PACAP), including patients aged 18 years or older with LAPC or mPC diagnosed between 2016 and 2023.^11^ Clinical data were obtained from the Netherlands *Cancer* Registry (NCR), including patient characteristics, tumor location, stage, and treatment characteristics. The NCR is a population-based registry collecting data on all newly diagnosed malignancies in the Netherlands. Notification sources are the Dutch Nationwide Pathology Databank (PALGA) and the Dutch National Hospital Care Registration (hospital admissions and ambulant diagnoses). Subsequently, trained NCR registrars routinely extract patient, tumor, and treatment information from medical records in all Dutch hospitals. Annual linkage with the Municipal Administrative Database, in which registers of all deceased or emigrated individuals are registered, provides information on vital status. Approval was obtained from the Privacy Review Board of the NCR and from the Scientific Committee of the Dutch Pancreatic *Cancer* Group (DPCG). According to the Central Committee on Research involving Human Subjects, this type of study does not require approval from an ethics committee in the Netherlands. The reporting of this study followed the Strengthening the Reporting of Observational Studies in Epidemiology (STROBE) guidelines.^14^ Patient-reported outcome measures (PROMs) were collected as part of the PACAP-PROMs initiative and linked to NCR data.^29^ Patients provided written informed consent to participate in PACAP and to link their patient-reported outcomes with clinical data from the NCR. The present analysis was performed retrospectively using pseudonymized, linked data.

### 2.2. Patient selection

Patients were included if tumor characteristics were available and if at least the baseline questionnaire was completed. After inclusion, patients were stratified into 2 groups: LAPC and mPC.

### 2.3. PROMs and pain classification

Patient-reported pain data were obtained through the EuroQol 5 Dimensions 5 Levels questionnaire (EQ-5D-5L), European Organization for Research and Treatment of *Cancer* Quality of Life Questionnaire-Core 30 (EORTC QLQ-C30), and the European Organization for Research and Treatment of *Cancer* Quality of Life Questionnaire- Pancreatic *Cancer* Module (26 items) (EORTC QLQ-PAN26). These are validated tools for assessing health-related quality of life in cancer patients, including disease-specific symptoms in pancreatic cancer.^21,23,27^

We selected 5 pain-related questions reflecting different dimensions of pain, which were labeled Q1 to Q5 for use in the analyses:

Q1: current pain (EQ-5D-5L, item 4),
Q2: pain during the past week (QLQ-C30, item 9),
Q3: interference of pain with daily activities (QLQ-C30, item 19),
Q4: back pain (QLQ-PAN26, item 4),
Q5: pain at night (QLQ-PAN26, item 3)

Each of the 5 pain-related questions was rated on a 5-point ordinal scale, although the exact wording differed across questionnaires. The EQ-5D-5L item assessed current pain with response options ranging from “no pain” to “extreme pain.” The EORTC QLQ-C30 and QLQ-PAN26 items used the scale “not at all,” “a little,” “quite a bit,” “very much,” and “extremely” to rate pain over the past week, pain interference, and location-specific pain.

Despite the variation in phrasing, all items reflect increasing levels of pain. For descriptive analyses, response distributions are presented per item. For regression analyses, the original ordered response categories were retained for the 2 main outcomes (“current pain” and “pain interference with daily activities”) and analyzed using ordinal mixed-effects models, thereby preserving clinically relevant differences in pain severity. Although these instruments are typically summarized into composite scores, we analyzed pain-related items separately to capture distinct dimensions of pain relevant to pancreatic cancer. Patients were enrolled at various stages of the disease trajectory, including before primary treatment, during treatment, or at progression/follow-up; for all analyses, the baseline assessment (T0) was used regardless of the timing of inclusion.

### 2.4. Outcomes and treatment classification

The primary outcome was the prevalence of pain at baseline and its evolution over time. Secondary analyses assessed the association of chemotherapy with “Current pain” and “Interference of pain with daily activities.” Chemotherapy was classified as a time-dependent exposure, using the recorded start and end dates of treatment. In addition, patients were grouped into 2 categories: those receiving best supportive care (BSC; no active cancer treatment at any time) and those receiving other treatment strategies. This treatment group included chemotherapy, radiotherapy, surgical interventions, or any combination. Only questionnaires completed after the start of therapy were included for the treatment group, whereas all questionnaires from the BSC group were retained.

### 2.5. Statistical methods

Descriptive statistics summarized baseline characteristics, treatment modalities, and pain outcomes across timepoints. Based on data distribution, between-group comparisons were performed using Pearson χ^2^ test, Fisher exact test, or the Mann–Whitney *U* test. Pain severity at baseline was presented using proportions of all 5 pain questions, and stratified for patients with LAPC and those with mPC. Differences were assessed using Pearson χ^2^ test or Fisher exact test. To assess temporal trends in pain, the proportion of patients reporting pain (binary: yes/no) at each time point (T0, T3, T6, T9, T12, and T18) was calculated for all 5 questions and stratified by tumor stage (LAPC vs mPC) or treatment group (BSC vs treatment). Patients were enrolled at various stages of the disease trajectory. To investigate the association between chemotherapy exposure and pain severity, 2 multivariable ordinal mixed-effects models were constructed for “Current pain” and “Interference with daily activities”. The original ordered response categories were used as dependent variables to preserve clinically relevant differences in pain severity. Fixed-effect covariates included chemotherapy exposure (a time-dependent fixed-effect covariate), radiotherapy exposure, and surgical resection (fixed covariates), timepoint, age, sex, tumor stage, and tumor location (head, body, tail, or other). Covariates were selected a priori based on their clinical relevance as potential confounders of the relationship between chemotherapy and pain. A random intercept for patient ID was included to account for within-subject clustering of repeated measures. The variable “timepoint” was entered as a categorical predictor, with T0 (baseline) set as the reference category and T12 and T18 combined as ≥T12 because of decreasing response rates at later follow-up. Odds ratios (ORs) and corresponding 95% confidence intervals (CIs) were estimated from the ordinal mixed-effects models. ORs below 1 indicate lower odds of being in a higher pain severity category, whereas ORs above 1 indicate higher odds of being in a higher pain severity category. Model fitting was performed using the glmmTMB package in R (version 4.5.0). Descriptive analyses were conducted in IBM SPSS Statistics (version 30).

## 3. Results

**Figure 1.**
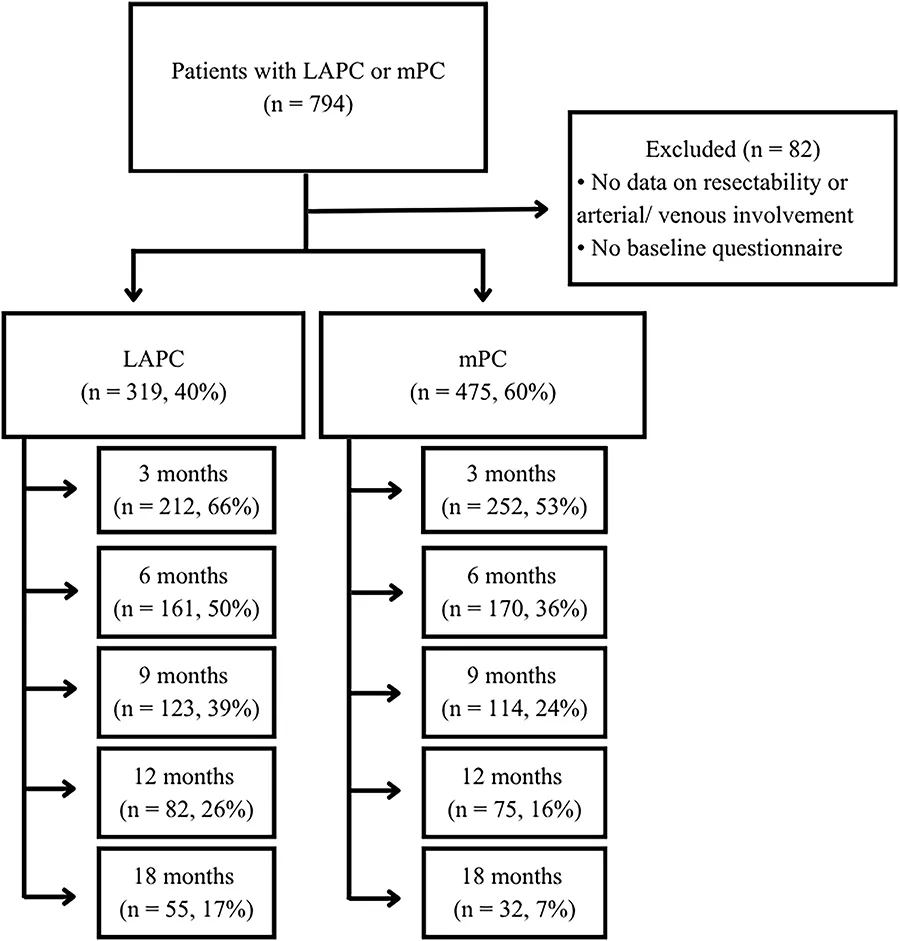
Flow diagram of patient selection. Overview of patient selection and follow-up. Patients were excluded based on incomplete tumor classification (ie, venous or arterial involvement) or missing baseline questionnaires. The number of patients who completed follow-up questionnaires at 3, 6, 9, 12, and 18 months is presented.

Overall, 876 patients were eligible for the study. Of these, 82 were excluded because of insufficient information regarding tumor stage or incomplete responses to the baseline questionnaire (Fig. 1). The final cohort included 794 patients, of whom 319 (40%) had LAPC and 475 (60%) had mPC. Baseline characteristics were comparable between the 2 groups (Table 1). The cohort comprised 52% male patients, with a median age of 65 years. Tumors were most frequently located in the head of the pancreas (55%). Among patients with metastatic disease, the liver was the most frequently affected site (46%). World Health Organization (WHO) performance status was 1 in 41% of patients, 0 in 38%, and 57% had no recorded comorbidities. Within the treatment group, 637 patients (80%) received chemotherapy, 61 (8%) received radiotherapy, and 96 (12%) underwent surgery; patients may have received more than one type of treatment. Chemotherapy alone was administered in 69% of mPC patients and 51% of those with LAPC. Best supportive care alone (ie, without any cancer-directed treatment) was provided in 23% of mPC patients and 12% of LAPC patients. The mean interval between diagnosis and completion of the baseline questionnaire was 62 days (95% CI 53.0-72.4), whereas the mean time to initiation of chemotherapy was 36 days (95% CI 33.8-37.8). The baseline questionnaire was completed in 408 patients (51%) before the start of cancer therapy and in 240 patients (30%) after treatment initiation. The remaining 146 patients (19%) received best supportive care only (Fig. 1). Longitudinal questionnaire completion rates were 58.4% (n = 464) at 3 months, 36.6% (n = 150) at 6 months, 29.8% (n = 237) at 9 months, 19.8% (n = 157) at 12 months, and 11.0% (n = 87) at 18 months (Fig. 1).

**Table 1:** Baseline characteristics of the population.

Variable	Total n = 794	LAPC n = 319	mPC n = 475	*P*
Age, mean (SD), y	65 (9.8)	66 (8.2)	65 (9.6)	0.016
Sex, n (%)				
Female	377 (48%)	150 (47%)	227 (48%)	0.832
BMI, median (IQR), kg/m^2^	24 (22-26)	24 (22-26)	24 (22-26)	0.953
Comorbidities (CCI)				0.179
0	426 (57%)	167 (54%)	269 (59%)	
1	225 (29%)	98 (31%)	127 (28%)	
>2	107 (14%)	46 (15%)	61 (13%)	
Missing	26 (3%)	8 (3%)	18 (4%)	
WHO performance status, n (%)				0.112
0	300 (38%)	133 (42%)	167 (36%)	
1	328 (41%)	224 (39%)	204 (43%)	
>2	31 (12%)	37 (11%)	54 (11%)	
Unknown or missing	75 (10%)	25 (8%)	50 (11%)	
Primary tumor location				**<0.001**
Head	437 (55%)	214 (67%)	223 (47%)	
Body	168 (21%)	64 (20%)	104 (22%)	
Tail	115 (15%)	17 (5%)	98 (21%)	
Other	84 (9%)	24 (8%)	50 (10%)	
Metastatic site, n (%)		—		—
Liver-only	215 (45%)		215 (45%)	
Lung-only	21 (4%)		21 (4%)	
Lymph-only	29 (6%)		29 (6%)	
Peritoneum-only	47 (10%)		47 (10%)	
Multiple	152 (32%)		152 (32%)	
Unknown or other	11 (3%)		11 (3%)	
Type of treatment, n (%)				**<0.001**
Best supportive care only*	146 (18%)	39 (12%)	107 (23%)	
Any kind of treatment	648 (82%)	280 (88%)	368 (78%)	
Chemotherapy	637 (80%)	276 (87%)	361 (76%)	
Radiotherapy	61 (8%)	43 (13%)	18 (4%)	
Surgery	96 (12%)	84 (26%)	12 (3%)	

Data are presented as mean ± SD, median with interquartile range (IQR), or number with corresponding percentage (%), as appropriate. Continuous variables were compared using the Mann–Whitney *U* test; categorical variables were analyzed using the χ2 test or Fisher exact test, where applicable. Bold values indicate statistically significant differences (p < 0.05). Percentages may not total 100% because of rounding. BMI, body mass index; LAPC, locally advanced pancreatic cancer; mPC, metastatic pancreatic cancer; WHO, World Health Organization.

* Patients may have received more than one type of treatment; percentages do not add up to 100%.

### 3.1. Pain at baseline

**Figure 2.**
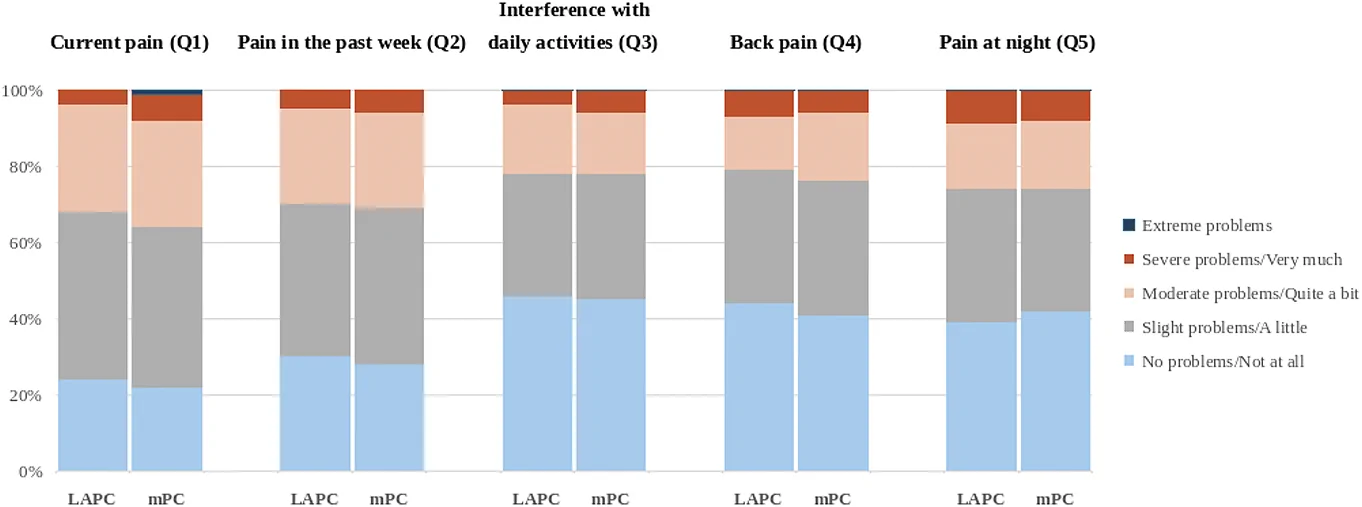
An overview of the 5 questions at baseline in patients with locally advanced and metastatic pancreatic cancer. Distribution of responses to 5 pain-related questions at baseline, irrespective of time since diagnosis or treatment initiation (n = 794). Pain was assessed using the following items: EQ-5D-5L Q4 (“Do you have pain or discomfort right now?”) (Q1), EORTC QLQ-C30 Q9 (“Have you had pain during the past week?”) (Q2), EORTC QLQ-C30 Q19 (“Did pain interfere with your daily activities?”) (Q3), EORTC QLQ-PAN26 Q4 (“Have you had pain in your back?”) (Q4), and EORTC QLQ-PAN26 Q3 (“Have you had pain at night?”) (Q5). Answer categories ranged from “not at all” to “very much’’ or “No problems” to “Extreme problems”. This figure offers a general overview of pain burden before stratification by treatment or time point.

At baseline, most patients in both the LAPC and mPC groups reported having pain, with most describing their symptoms as slight (Fig. 2, supplemental digital content, table S1, http://links.lww.com/PAIN/C540). Although patients with mPC numerically reported more severe complaints related to pain, compared with those with LAPC, there were no statistically significant differences in pain severity between the groups across any of the 5 pain-related questions (all *P* > 0.05).

### 3.2. Longitudinal outcome of pain

**Figure 3.**
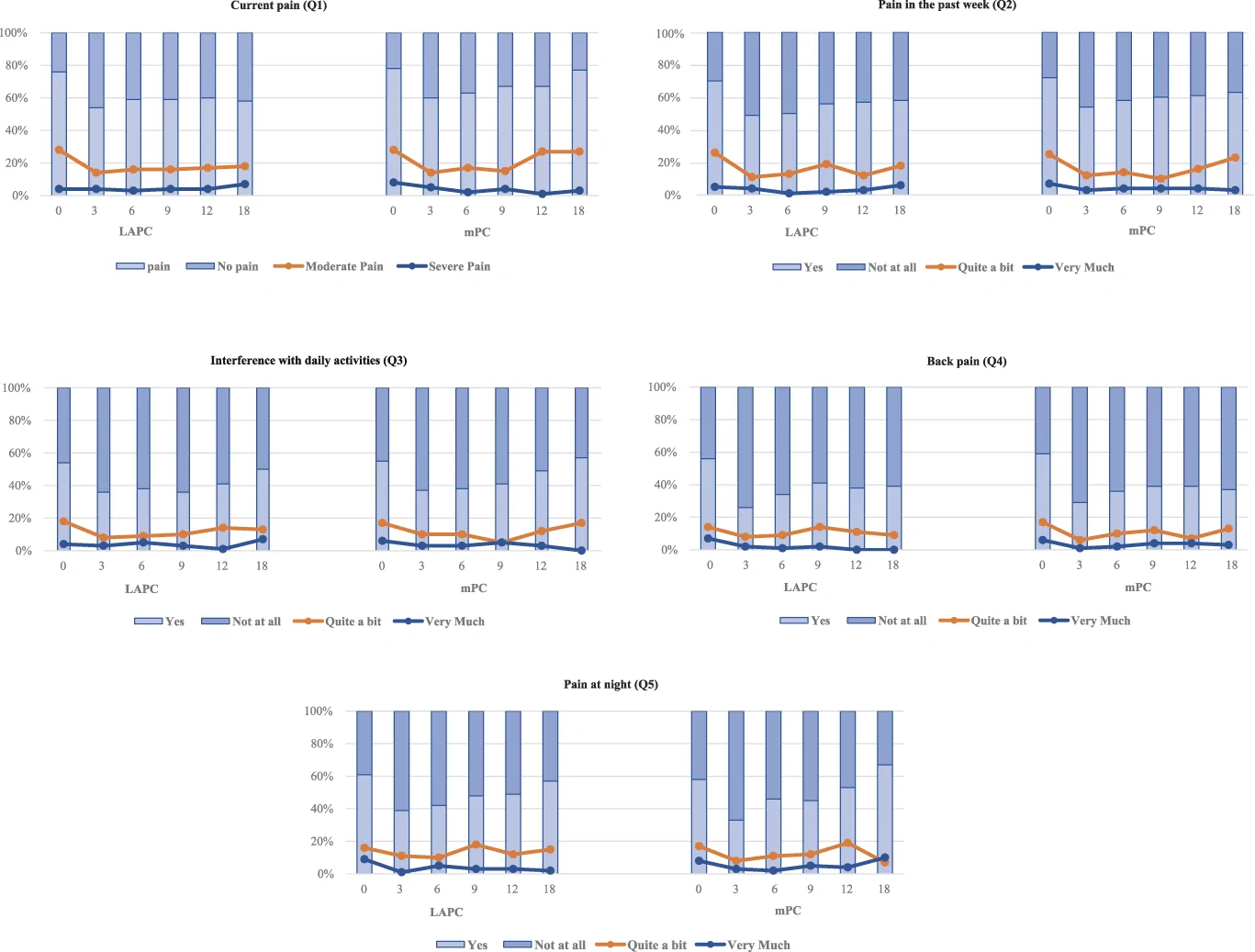
Overview of pain over time (in months) in 5 different questions concerning pain, stratified for locally advanced and metastatic pancreatic cancer. Overview of the 5 different pain questions at baseline and follow-up, regardless of time of diagnosis and time of treatment initiation. Questions: EQ-5D-4 (*Do you experience pain right now?*) (Q1), EORTC-QLQ-C309 (*Have you experienced pain in the last week?*) (Q2), EORTC-QLQ-C3019 (*Did the pain influence your daily activities?*) (Q3), EORTC-QLQ-PAN4 (*Do you have back pain?*) (Q4), and the EORTC-QLQ-PAN3 (*Do you have pain at night?*) (Q5).

Figure 3 provides an overall illustration of pain trajectories over time for all patients, regardless of when they were diagnosed or started treatment (supplemental digital content, table S2 provides the absolute numbers, http://links.lww.com/PAIN/C540). The figure illustrates the general trends in pain reporting at various time points, without stratification by therapy status. A decrease in pain prevalence was observed at 3 months compared with baseline, with moderate and severe pain levels remaining consistently low throughout the follow-up period. The most considerable reduction in reported pain was observed in responses to the item addressing pain in the week preceding the questionnaire (Q2). No statistically significant differences were found between the LAPC and mPC groups at any time point.

### 3.3. Pain stratified by treatment group

**Figure 4.**
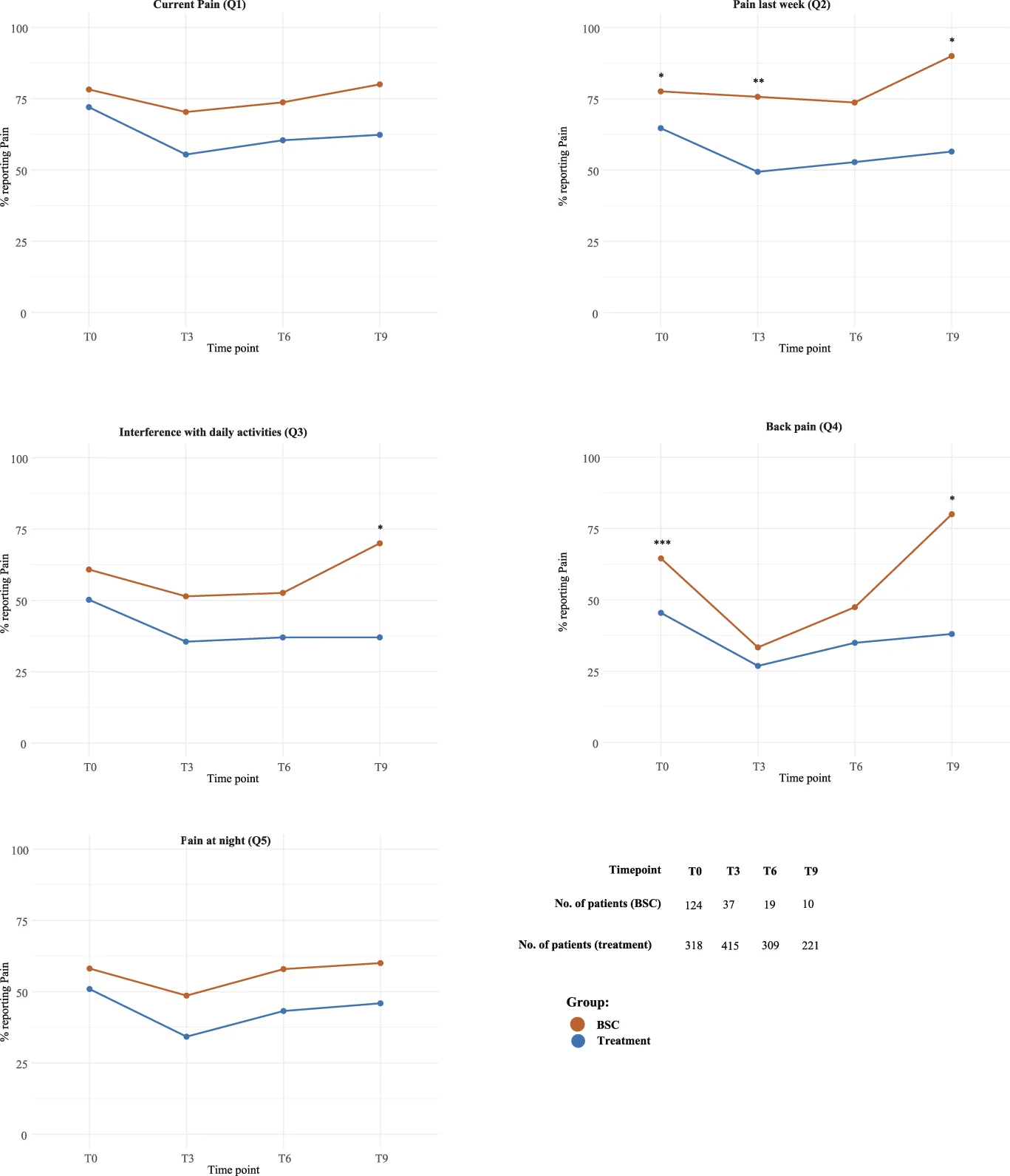
Overview of pain over time (in months) in 5 different questions concerning pain, stratified for BSC and cancer treatment (radiotherapy, surgery, and/or chemotherapy). Overview of the 5 different pain questions over time stratified for patients receiving BSC vs patients who receive treatment. Questions: EQ-5D-4 (*Do you experience pain right now?*) (Q1), EORTC-QLQ-C309 (*Have you experienced pain in the last week?*) (Q2), EORTC-QLQ-C3019 (*Did the pain influence your daily activities?*) (Q3), EORTC-QLQ-PAN4 (*Do you have back pain?*) (Q4), and the EORTC-QLQ-PAN3 (*Do you have pain at night?*) (Q5). Treatment: Radiotherapy, surgery, or chemotherapy. Absolute numbers and values are reported in Supplemental digital content (see Table S3, http://links.lww.com/PAIN/C540). Asterisks indicate statistically significant differences between groups at each time point (χ^2^ or Fisher exact test): *P <0.05, **P <0.01, ***P <0.001.

Figure 4 and Supplemental digital content (see Table S3, http://links.lww.com/PAIN/C540) compare patient-reported pain between patients receiving best supportive care (BSC, n = 146; 18%) and those receiving anticancer treatment (n = 648; 82%).

Across all 5 pain domains, patients in the BSC group reported a higher prevalence of pain compared with those receiving anticancer therapy. The difference was most pronounced for the item “pain in the past week” (Q2), reported by 78% of BSC patients at baseline (T0) vs 65% in the treatment group (*P* = 0.012), and persisting at 3 months (76% vs 49%, *P* = 0.004) and 9 months (90% vs 57%, *P* = 0.048). Back pain (Q4) was also more common among BSC patients at baseline (65% vs 45%, *P* < 0.001) and at 9 months (80% vs 38%, *P* = 0.016). Pain interfering with daily functioning (Q3), current pain (Q1), and pain at night (Q5) consistently showed higher values in the BSC group across all timepoints.

### 3.4. Association of chemotherapy with pain

The results of the multivariable ordinal mixed-effects models are shown in Tables 2 and 3. In both models, exposure to chemotherapy was statistically associated with a lower odds ratio of being in a higher pain severity category. For current pain, the OR among patients receiving chemotherapy was 0.51 (95% CI 0.37-0.70; *P* ≤ 0.001). A similar association was observed for interference with daily activities, where chemotherapy exposure was associated with an OR of 0.44 (95% CI 0.31-0.63; *P* < 0.001).

**Table 2:** Ordinal mixed-effects analysis of the influence of chemotherapy on current pain.

Variable	Odds ratio	95% CI interval	*P*
Time-fixed covariates			
Radiotherapy	0.77	0.45-1.29	0.32
Surgery	0.63	0.39-1.01	**0.05**
Time			
Baseline (reference)	—	—	—
T3	0.62	0.44-0.87	**0.005**
T6	0.70	0.50-0.96	**0.029**
T9	0.60	0.43-0.83	**0.002**
≥T12	0.78	0.56-1.08	0.13
Age	0.81	0.70-0.94	**0.007**
Sex			
Female (reference)	—	—	—
Male	0.66	0.49-0.88	**0.005**
Tumor stage			
LAPC (reference)	—	—	—
mPC	1.01	0.73-1.41	0.93
Tumor location			
Body (reference)	—	—	—
Head	0.68	0.47-1.00	**0.05**
Tail	0.90	0.56-1.24	0.76
Other	0.69	0.38-1.24	0.21
WHO performance status			
WHO 0-1 (reference)	—	—	—
WHO 2	1.88	1.10-3.19	**0.02**
WHO 3-4	4.43	1.65-11.87	**0.003**
Time-dependent covariate			
Chemotherapy	0.51	0.37-0.70	**<0.001**

Ordinal mixed-effect model with random intercepts per patient (glmmTMB) to account for repeated measures. Odds ratios (ORs), 95% confidence intervals (CIs), and *P*-values are shown. Pain was modeled as a binary outcome (1 = presence of pain). The number of patients with available ‘‘Current Pain’’ data was 780 at T0, 457 at T3, 329 at T6, 233 at T9, and 242 at ≥T12. Missing outcome values were excluded only for the respective time point, whereas other measurements from the same patient were retained. Bold values indicate statistical significance at *P* < 0.05.

**Table 3:** Ordinal mixed-effects analysis of the influence of chemotherapy on the interference of pain on daily activities.

Variable	Odds ratio	95% CI interval	*P*
Time-fixed covariates			
Radiotherapy	0.94	0.55-1.59	0.81
Surgery	0.49	0.30-0.79	**0.003**
Time			
Baseline (reference)	—	—	—
T3	0.83	0.58-1.18	0.29
T6	0.84	0.60-1.18	0.34
T9	0.69	0.48-0.99	**0.04**
≥T12	1.18	0.83-1.67	0.36
Age	0.72	0.63-0.84	<**0.001**
Sex			
Female (reference)	—	—	—
Male	0.60	0.45-0.81	**<0.001**
Tumor stage			
LAPC (reference)	—	—	—
mPC	0.81	0.58-1.13	0.21
Tumor location			
Body (reference)	—	—	—
Head	0.94	0.64-1.37	0.73
Tail	1.10	0.66-1.83	0.73
Other	1.02	0.56-1.84	0.96
WHO performance status			
WHO 0-1 (reference)	—	—	—
WHO 2	2.58	1.53-4.35	**<0.001**
WHO 3-4	5.56	2.16-14.35	**<0.001**
Time-dependent covariates			
Chemotherapy	0.44	0.31-0.63	**<0.001**

Ordinal mixed-effect model with random intercepts per patient (glmmTMB) to account for repeated measures. Odds ratios (ORs), 95% confidence intervals (CIs), and *P*-values are shown. Pain was modeled as a binary outcome (1 = presence of pain). The number of patients with available ‘‘Current Pain’’ data was 784 at T0, 457 at T3, 326 at T6, 229 at T9, and 238 at ≥T12. Missing outcome values were excluded only for the respective time point, whereas other measurements from the same patient were retained. Bold values indicate statistical significance at *P* < 0.05.

Over time, the odds ratio of being in a higher current pain category were lower at 3, 6, and 9 months compared with baseline (T3: OR 0.62, 95% CI 0.44-0.87; T6: OR 0.70, 95% CI 0.50-0.96; and T9: OR 0.60, 95% CI 0.43-0.83; all *P* < 0.05) but not at ≥12 months. For interference with daily activities, a statistically significant reduction compared with baseline was observed only at 9 months (T9: OR 0.69, 95% CI 0.48-0.99, *P* = 0.04).

Older age and male sex were associated with a lower odds ratio of being in a higher pain severity category in both models (current pain: OR 0.81, 95% CI 0.70-0.94, *P* = 0.007 and OR 0.66, 95% CI 0.49-0.88, *P* = 0.005; interference with daily activities: OR 0.72, 95% CI 0.62-0.84, *P* ≤ 0.001; and OR 0.60, 95% CI 0.45-0.81, *P* ≤ 0.001). Worse WHO performance status was associated with a significantly higher odds ratio of being in a higher pain severity category for both current pain and pain interference with daily activities. For current pain, WHO 2 was associated with an OR of 1.88 (95% CI 1.10-3.19, *P* = 0.020), and WHO 3 to 4 with an OR of 4.43 (95% CI 1.65-11.87, *P* = 0.003). For pain interference, WHO 2 was associated with an OR of 2.58 (95% CI 1.53-4.35, *P* ≤ 0.001), and WHO 3 to 4 with an OR of 5.56 (95% CI 2.16-14.35, *P* < 0.001). No other covariates, including tumor stage, tumor location, or radiotherapy, were associated with either outcome.

## 4. Discussion

This first nationwide to assess the longitudinal course of pain in advanced pancreatic cancer found pain to be prevalent at baseline across multiple domains, most often with mild to moderate severity. Although no significant differences were observed between patients with LAPC and mPC, those receiving best supportive care consistently reported more pain than those receiving anticancer treatment. Pain generally declined over time, particularly during the first 3 months after diagnosis, in multivariable mixed-effects models; chemotherapy exposure and time since diagnosis were statistically associated with a lower likelihood of reporting pain and its interference with daily functioning.

Pain remains a highly prevalent and burdensome symptom in patients with cancer.^6,62^ Our findings are in line with prior studies reporting high rates of pain among patients with cancer, particularly those with pancreatic malignancies.^6,15,22,28,35,36,46,50,51,53,57,58,61,62^ Although most prior studies assess pain as part of broader QoL outcomes or treatment evaluations, longitudinal and population-based data focusing specifically on pain in pancreatic cancer are limited. Our study contributes to this evidence base by using repeated patient-reported outcomes to provide a more granular understanding of how pain evolves and concerning treatment exposure across subgroups.

We observed high rates of pain in both the LAPC and mPC groups, with no major clinical or statistically significant differences. Previous studies have suggested that pain prevalence may be associated with tumor stage.^28,61^ For instance, Damm et al. found that while the frequency of pain symptoms increased with disease progression, the severity of pain remained comparable across stages.^15^ Several factors may explain the absence of clear group differences in our study. First, our cohort largely consisted of relatively fit patients; only 1 in 5 received BSC alone, whereas national data suggest that the majority of patients do not receive tumor-directed therapy.^30,66^ This selection bias may have reduced variability in symptom burden. Second, attrition because of early mortality may have been more frequent in the mPC group, which could have attenuated differences over time. However, detailed data on survival per time point were not available. Third, the pain-relieving effect of chemotherapy, as supported by our multivariable models, could help explain why pain levels become more similar over time across both groups.

We found that pain prevalence declined during the first 3 months after diagnosis, followed by a slight increase at later time points. This pattern may reflect the initial benefit of anticancer therapy, combined with attrition of patients with the highest symptom burden because of early progression or death. At the same time, local interventions such as surgery or radiotherapy, more common in the LAPC group, may also have influenced these trajectories. For example, radiotherapy has been shown to provide a rapid and clinically meaningful reduction in pain severity in pancreatic cancer.^39,47,60^ However, the evidence for long-term pain relief remains mixed.^2,5,31^ Importantly, although anticancer treatment may alleviate symptoms, overall survival in advanced pancreatic cancer remains limited. Our findings should therefore be interpreted in the context of a poor prognosis and the likely underrepresentation of untreated patients, who may experience an even greater symptom burden than observed in this cohort.

In addition to persistent background pain, patients with advanced pancreatic cancer may experience BTcP, characterized by transient pain exacerbations despite controlled baseline pain. Because the applied PROMs assess overall pain burden rather than episodic fluctuations, BTcP may represent an additional symptom component not fully captured in this study.^16,55^ Pain trajectories may also be influenced by factors beyond tumor biology or treatment, including psychological and social dimensions. A growing body of evidence highlights the interplay between cancer-related pain and psychological distress, including depression, anxiety, fear, and worry, all of which may modulate patients' perception and reporting of pain symptoms.^9,32^ Pancreatic cancer is among the cancer types most strongly associated with depression.^41^ In a study by Arora et al., 36.7% of pancreatic cancer patients reported pain, and 32.8% had anxiety, compared with 17% and 9.2%, respectively, in patients without pain.^1^ These findings suggest that variations in psychological well-being may contribute to differences in the longitudinal experience of pain.

Chemotherapy plays a central role in the treatment of both LAPC and mPC, offering modest survival benefits.^3,34,59,64,65^ Our findings suggest that it may also alleviate pain and improve functional wellbeing, supporting earlier reports of improved QoL with systemic therapy.^8,12,22,24^ By specifically examining multiple dimensions of pain over time, our study adds to this evidence and highlights the symptomatic benefits of chemotherapy. The type of chemotherapy regimen may influence the pain trajectory. For example, FOLFIRINOX, a combination of oxaliplatin, irinotecan, fluorouracil, and leucovorin, has been associated with delayed deterioration in pain and quality of life compared with gemcitabine monotherapy.^19,24,37,63^ However, these findings are derived from highly selected populations and do not routinely evaluate pain as a primary outcome.

By contrast, patients receiving best supportive care alone consistently reported higher pain levels across all 5 domains, aligning with earlier findings of impaired QoL in this group.^10^ These results underscore the importance of timely pain assessment in shared decision-making and counseling, including for patients not eligible for, or not willing to undergo, systemic therapy. EU-wide initiatives such as the MyPath program, which promote patient-centered care and structured digital symptom monitoring, may facilitate the integration of proactive pain management into routine palliative care.^56^ Importantly, physical pain is only one dimension of palliative care, alongside psychological, social, and existential needs. Evidence from advanced cancer populations supports early integrated palliative care to improve patient-centered outcomes, and this approach may be particularly relevant for patients with advanced pancreatic cancer.^26,45^

However, the consistently higher pain levels observed in patients receiving best supportive care raise concerns about whether current palliative strategies adequately address symptom burden in this subgroup. This finding suggests that more structured and proactive pain management is needed, even in the absence of tumor-directed therapy, and calls into question whether current interpretations of “best” supportive care truly reflect optimal palliative practice.

A key strength is the large nationwide cohort with longitudinal patient-reported outcome data. This study also has several limitations. First, because of disease progression and clinical deterioration, including death, patients with a higher symptom burden may have been more likely to be lost to follow-up, which may have led to an underestimation of pain severity over time. Missing data are therefore likely to be informative rather than missing at random, representing a limitation of the applied mixed-effects models. Second, the study population was treatment-heavy, with over 60% of patients receiving chemotherapy and nearly half of the LAPC group undergoing local therapy. As many patients with advanced pancreatic cancer do not receive tumor-directed treatment in routine clinical practice, this cohort may represent a relatively fit subgroup. This may have limited the generalizability of our findings and may have contributed to both underestimation of pain severity and overestimation of treatment-associated improvements in pain. Third, detailed data on pain management were not available. Given that up to 75% of pancreatic cancer patients receive opioids, and that both pharmacological and interventional treatments can substantially reduce pain burden, unmeasured variation in pain management may have influenced our findings.^15,17,18,43,48,52^ This includes the observed association between chemotherapy exposure and lower pain severity. In addition, patients receiving best supportive care may have had different or more limited access to structured pain management, which may have contributed to between-group differences.

Fourth, the PACAP cohort was not designed with pain as a dedicated primary endpoint. Although the applied PROMs capture multiple dimensions of pain, including interference with daily activities and pancreatic cancer-specific symptoms, no detailed information on pain coping, dynamic pain patterns such as breakthrough cancer pain, or a 0 to 10 numeric rating scale was available. Future studies specifically targeting pain outcomes may benefit from incorporating dedicated pain measurement tools alongside multidimensional PROMs. Finally, response rates decreased over time, particularly at later follow-up timepoints, resulting in smaller subgroup sizes and less stable estimates in longitudinal analyses. Findings at these later timepoints should therefore be interpreted with caution.

This is the first nationwide study to provide a detailed longitudinal characterization of patient-reported pain in advanced pancreatic cancer. Most patients reported pain, affecting multiple domains of daily life in advanced disease. Chemotherapy exposure was associated with a lower probability of being in a higher pain severity category, whereas patients receiving best supportive care experienced higher pain burdens. These findings underscore the importance of timely recognition and management of pain from diagnosis through follow-up. Given the limited life expectancy associated with advanced pancreatic cancer, pain control should be integrated within comprehensive palliative care that provides continuous, multidisciplinary support to patients and their families throughout the disease trajectory. Further prospective studies with standardized pain assessments and structured interventions targeting pain reduction are warranted to optimize symptom management in this population.

## Conflict of interest statement

J. W. Wilmink: Consulting/advisor MSD, Servier, Novartis, AstraZeneca, Nordic. Research funding is given to the institutions Servier, Nordic, and MSD; all outside the submitted work. M. G. Besselink has been rewarded by KWF Dutch Cancer Society (#2021-2/14106) and Deltaplan Alvleesklierkanker (#2022-3/WOO 2102) for the Dutch PREOPANC-4 trial on multidisciplinary management of locally advanced pancreatic cancer (NCT05524090); by Intuitive Surgical for the DIPLOMA-2 trial on minimally invasive pancreatoduodenectomy (ISRCTN27483786); by Ethicon and Intuitive for the European quality registry on minimally invasive pancreatic surgery; and by Intuitive for the European LEARNBOT training program for robot-assisted pancreatoduodenectomy; all outside this submitted work. J. de Vos-Geelen has received institutional research funding from Servier; all outside the submitted work. H. W. M. van Laarhoven has received research funding or medication/material supply from: AMGEN, Auristone, BMS, Incyte, Merck, ORCA, Servier. Consultant/advisory role: AMGEN, Amphera, Astellas, AstraZeneca, Beigene, BMS, Boehringer, Daiichy, MSD, MyeloidTx. Speaker role: AstraZeneca, BMS, Congress Care, Daiichy, Medtalks, Uitgeverij JAAP, Travel Congress Management. The remaining authors have no conflicts of interest to declare.

## Supplementary Material

## Appendix A. Supplemental digital content

Supplemental digital content associated with this article can be found online at http://links.lww.com/PAIN/C540.
