# Supplementary material for: Pain prevalence and intensity in advanced pancreatic cancer: a nationwide cohort study
Source: Pain. 2026 Jul 6;167(9):2216–26. doi: 10.1097/j.pain.0000000000004059 (PMC13528902; doi:10.1097/j.pain.0000000000004059)
Supplement: Supplementary file 1 [file jop-167-2216-s001.pdf]

Supplementary material

Supplementary table S1 I Baseline values for the five pain questions (T0)

	LAPC		mPC		P-value
**Current pain (Q1)**					
No problems	75	(24%)	104	(22%)	0.234
Slight problems	138	(44%)	196	(42%)	
Moderate problems	87	(28%)	128	(28%)	
Severe problems	13	(4%)	35	(7%)	
Extreme problems	1	(0%)	3	(1%)	
Missing	5	(2%)	9	(2%)	
**Pain in the past week (Q2)**					
Not at all	94	(30%)	129	(28%)	0.596
A little	126	(40%)	190	(41%)	
Quite a bit	81	(25%)	114	(25%)	
Very much	17	(5%)	32	(7%)	
Missing	1	(0%)	10	(2%)	
**Interference with daily activities (Q3)**					
Not at all	147	(46%)	209	(45%)	0.631
A little	101	(32%)	154	(33%)	
Quite a bit	56	(18%)	77	(16%)	
Very much	13	(4%)	27	(6%)	
Missing	2	(1%)	8	(2%)	
**Back pain (Q4)**					
Not at all	137	(44%)	191	(41%)	0.366
A little	110	(35%)	165	(35%)	
Quite a bit	44	(14%)	81	(18%)	
Very much	21	(7%)	29	(6%)	
Missing	7	(2%)	9	(2%)	
**Pain at night (Q5)**					
Not at all	124	(39%)	197	(42%)	0.498
A little	110	(35%)	151	(32%)	
Quite a bit	52	(17%)	81	(18%)	
Very much	29	(9%)	37	(8%)	
Missing	4	(1%)	10	(2%)	

Percentages may not sum to 100% due to rounding. Categorical variables were examined using the chi-squared test or Fisher's exact test, as appropriate.

Supplementary table S2 I Values over time for the five pain questions (T0)

	LAPC		mPC		P-value
**Current pain (Q1)**					
**Baseline**	**314**		**466**		**0.610**
Pain[A]	239	(76%)	362	(78%)	
Moderate	87	(28%)	128	(28%)	
Severe	14	(4%)	38	(8%)	
**3 Months**	**207**		**250**		**0.199**
Pain[A]	111	(54%)	149	(60%)	
Moderate	28	(14%)	36	(14%)	
Severe	9	(4%)	12	(5%)	
**6 Months**	**160**		**169**		**0.396**
Pain[A]	94	(59%)	107	(63%)	
Moderate	26	(16%)	28	(17%)	
Severe	5	(3%)	4	(2%)	
**9 Months**	**120**		**113**		**0.210**
Pain[A]	71	(59%)	76	(67%)	
Moderate	19	(16%)	17	(15%)	
Severe	5	(4%)	4	(4%)	
**12 Months**	**82**		**75**		**0.370**
Pain[A]	49	(60%)	50	(67%)	
Moderate	14	(17%)	20	(27%)	
Severe	3	(4%)	1	(1%)	
**18 Months**	**55**		**30**		**0.088**
Pain[A]	32	(58%)	23	(77%)	
Moderate	10	(18%)	8	(27%)	
Severe	4	(7%)	1	(3%)	
**Pain in the past week (Q2)**					
**Baseline**	**318**		**465**		**0.580**
Pain[B]	224	(70%)	336	(72%)	
Quite a bit	81	(26%)	114	(25%)	
Very much	17	(5%)	32	(7%)	
**3 Months**	**208**		**250**		**0.384**
Pain[B]	103	(49%)	134	(54%)	
Quite a bit	22	(11%)	31	(12%)	
Very much	8	(4%)	7	(3%)	
**6 Months**	**160**		**170**		**0.164**
Pain[B]	80	(50%)	98	(58%)	
Quite a bit	20	(13%)	23	(14%)	
Very much	3	(1%)	6	(4%)	
**9 Months**	**120**		**113**		**0.502[A]**
Pain[B]	67	(56%)	68	(60%)	
Quite a bit	23	(19%)	11	(10%)	
Very much	2	(2%)	5	(4%)	
**12 Months**	**81**		**75**		**0.564**
Pain[B]	46	(57%)	46	(61%)	
Quite a bit	10	(12%)	12	(16%)	
Very much	2	(3%)	3	(4%)	
**18 Months**	**55**		**30**		**0.643**
Pain[B]	32	(58%)	19	(63%)	
Quite a bit	10	(18%)	7	(23%)	
Very much	3	(6%)	1	(3%)	
**Interference with daily activities (Q3)**					

**Baseline**	**317**		**467**		**0.655**
Pain[B]	170	(54%)	258	(55%)	
Quite a bit	56	(18%)	77	(17%)	
Very much	13	(4%)	27	(6%)	
**3 Months**	**209**		**248**		**0.871**
Pain[B]	76	(36%)	92	(37%)	
Quite a bit	16	(8%)	25	(10%)	
Very much	7	(3%)	7	(3%)	
**6 Months**	**157**		**169**		**0.949**
Pain[B]	60	(38%)	64	(38%)	
Quite a bit	14	(9%)	17	(10%)	
Very much	7	(5%)	5	(3%)	
**9 Months**	**117**		**112**		**0.421**
Pain[B]	42	(36%)	46	(41%)	
Quite a bit	12	(10%)	5	(5%)	
Very much	3	(3%)	6	(5%)	
**12 Months**	**80**		**74**		**0.356**
Pain[B]	33	(41%)	36	(49%)	
Quite a bit	11	(14%)	9	(12%)	
Very much	1	(1%)	2	(3%)	
**18 Months**	**54**		**30**		**0.588**
Pain[B]	27	(50%)	17	(57%)	
Quite a bit	7	(13%)	5	(17%)	
Very much	4	(7%)	0	(0%)	
**Back pain (Q4)**					
**Baseline**	**312**		**466**		**0.418**
Pain[B]	175	(56%)	275	(59%)	
Quite a bit	44	(14%)	81	(17%)	
Very much	21	(7%)	29	(6%)	
**3 Months**	**207**		**246**		**0.452**
Pain[B]	54	(26%)	72	(29%)	
Quite a bit	16	(8%)	15	(6%)	
Very much	4	(2%)	2	(1%)	
**6 Months**	**158**		**170**		**0.664**
Pain[B]	54	(34%)	62	(36%)	
Quite a bit	14	(9%)	17	(10%)	
Very much	2	(1%)	3	(2%)	
**9 Months**	**118**		**113**		**0.787**
Pain[B]	48	(41%)	44	(39%)	
Quite a bit	16	(14%)	13	(12%)	
Very much	2	(2%)	4	(4%)	
**12 Months**	**81**		**74**		**0.907**
Pain[B]	31	(38%)	29	(39%)	
Quite a bit	9	(11%)	5	(7%)	
Very much	0	(0%)	3	(4%)	
**18 Months**	**54**		**30**		**0.841**
Pain[B]	21	(39%)	11	(37%)	
Quite a bit	5	(9%)	4	(13%)	
Very much	0	(0%)	1	(3%)	
**Pain at night (Q5)**					
**Baseline**	**315**		**466**		**0.418**
Pain[B]	191	(61%)	269	(58%)	
Quite a bit	52	(16%)	81	(17%)	
Very much	29	(9%)	37	(8%)	

**3 Months**	**209**		**249**		**0.161**
Pain[B]	82	(39%)	82	(33%)	
Quite a bit	23	(11%)	21	(8%)	
Very much	3	(1%)	7	(3%)	
**6 Months**	**160**		**169**		**0.505**
Pain[B]	68	(42%)	78	(46%)	
Quite a bit	16	(10%)	19	(11%)	
Very much	8	(5%)	4	(2%)	
**9 Months**	**119**		**113**		**0.673**
Pain[B]	57	(48%)	51	(45%)	
Quite a bit	21	(18%)	13	(12%)	
Very much	3	(3%)	6	(5%)	
**12 Months**	**81**		**74**		**0.680**
Pain[B]	40	(49%)	39	(53%)	
Quite a bit	10	(12%)	14	(19%)	
Very much	2	(3%)	3	(4%)	
**18 Months**	**54**		**30**		**0.405**
Pain[B]	31	(57%)	20	(67%)	
Quite a bit	8	(15%)	2	(7%)	
Very much	1	(2%)	3	(10%)	

Only the percentage of pain is reported. Percentages may not sum to 100% due to rounding. Categorical variables were examined using the chi-squared test or Fisher's exact test, as appropriate.

Supplementary table S3 I Values over time for the five pain questions, stratified for BSC and treatment

Question	Time	% pain BSC	% pain treatment	p-value	q-value
**Current pain (Q1)**					
	T0	78% (n=124)	72% (n=318)	0.225	0.225
	T3	70% (n=37)	55% (n=415)	0.116	0.116
	T6	74% (n=19)	60% (n=308)	0.362	0.362
	T9	80% (n=10)	62% (n=223)	0.331	0.331
**Pain in the past week (Q2)**					
	T0	78% (n=125)	65% (n=317)	**0.012**	**0.012**
	T3	76% (n=37)	49% (n=415)	**0.004**	**0.004**
	T6	74% (n=19)	53% (n=309)	0.124	0.124
	T9	90% (n=10)	57% (n=223)	**0.048**	**0.048**
**Interference with daily activities (Q3)**					
	T0	61% (n=125)	50% (n=317)	0.056	0.056
	T3	51% (n=37)	36% (n=414)	0.082	0.082
	T6	53% (n=19)	37% (n=305)	0.265	0.265
	T9	70% (n=10)	37% (n=219)	**0.047**	**0.047**
**Back pain (Q4)**					
	T0	65% (n=124)	45% (n=313)	**0.000**	**0.000**
	T3	33% (n=36)	267% (n=411)	0.514	0.514
	T6	47% (n=19)	35% (n=307)	0.390	0.390
	T9	80% (n=10)	38% (n=221)	**0.016**	**0.016**
**Pain at night (Q5)**					
	T0	58% (n=124)	51% (n=316)	0.215	0.215
	T3	49% (n=37)	34% (n=415)	0.114	0.114
	T6	58% (n=19)	43% (n=308)	0.310	0.310
	T9	60% (n=10)	46% (n=222)	0.521	0.521

Only the percentage of pain is reported. Percentages may not sum to 100% due to rounding. Treatment: Radiotherapy, chemotherapy, and radiotherapy. Categorical variables were examined using the chi-squared test or Fisher's exact test, as appropriate. Abbreviations: BSC; Best Supportive Care.
